# A Shared Decision-Making Approach to Telemedicine: Engaging Rural Patients in Glycemic Management

**DOI:** 10.3390/jcm5110103

**Published:** 2016-11-17

**Authors:** Michelle L. Griffith, Linda Siminerio, Tammie Payne, Jodi Krall

**Affiliations:** 1 Department of Medicine, Division of Endocrinology and Metabolism, University of Pittsburgh, Pittsburgh, PA 15213, USA; griffithml@upmc.edu (M.L.G.); similx@upmc.edu (L.S.); 2 UPMC Bedford Memorial Hospital, Everett, PA 15537, USA; paynetm@upmc.edu; 3 University of Pittsburgh Diabetes Institute, Pittsburgh, PA 15213, USA

**Keywords:** telemedicine, diabetes, shared decision-making, team-based model

## Abstract

Telemedicine can connect specialist health care providers with patients in remote and underserved areas. It is especially relevant in diabetes care, where a proliferation of treatment options has added further complexity to the care of an already complex, highly prevalent disease. Recent developments in health reform encourage delivery systems to use team-based models and engage patients in shared decision-making (SDM), where patients and providers together make health care decisions that are tailored to the specific characteristics and values of the patient. The goal of this project was to design, integrate, and evaluate a team-based, SDM approach delivered to patients with diabetes in a rural community, building upon the previously established telemedicine for reach, education, access, and treatment (TREAT) model. Patients in this feasibility study demonstrated improvement in hemoglobin A1c values, and reported better understanding of diabetes. Providers reported the SDM aids increased cohesion among team members (including patients) and facilitated patient education and behavioral goal setting. This project demonstrated that SDM could be integrated into the workflow of a telemedicine team visit with good provider and patient satisfaction.

## 1. Introduction

Diabetes is complex and highly prevalent, and under-served rural communities carry a disproportionate burden of the disease. Studies have shown that patients in rural communities unfortunately do not receive the same number and type of chronic care services, including diabetes services, as their urban counterparts [1]. Telemedicine, the application of telecommunications technology used to connect providers and patients who are geographically separated, is being explored in an effort to reach remote and underserved areas [2,3]. Telemedicine approaches are particularly relevant for diabetes. Optimal diabetes care requires active patient self-management, including dietary monitoring and self-monitoring of blood glucose along with medication adjustments guided by a health care provider. In fact, diabetes has been reported by primary care providers (PCPs), who provide the majority of diabetes care [4,5,6], to be more difficult to manage given the limited time for visits [7,8]. A rapid increase in novel treatment options in recent years adds to the complexity of diabetes care and increases the demand for specialty expertise and team-based services to complement primary care.

Despite progress of available therapies, patients can still have challenges in meeting target goals [9] and experience distress related to their treatment plan [10,11,12]. The influx of new and sophisticated therapies increases the importance of methods, such as shared decision-making (SDM), to facilitate communication about options and engage patients as active participants in their diabetes management decisions. SDM encourages providers and patients to collaboratively negotiate health-related decisions, taking into account scientific evidence as well as patient needs and preferences [13,14]. Decision aids, such as brochures or graphics, can be used by health care professionals to introduce patients to treatment options, outcomes, risks and benefits. While such aids are primarily used to compare various treatment options, they can also be used to familiarize patients with models of care and key disease management concepts. Within the context of diabetes, SDM and team-based models have been shown to be effective elements in improving diabetes self-management behaviors and outcomes [15,16].

Using a team-based approach delivered via the telemedicine for reach, education, access and treatment (TREAT) intervention, investigators at the University of Pittsburgh Medical Center (UPMC) demonstrated improvement in diabetes clinical outcomes, including improvement in hemoglobin A1c (HbA1c), along with improvements in patient self-care, empowerment, reduced diabetes distress and high provider/patient satisfaction [17,18,19]. The TREAT model includes an endocrinologist providing consultation via videoconferencing and a nurse diabetes educator (DE) at the patient’s side, who provides associated diabetes self-management education and support. The physician and diabetes educator are both integral parts of the TREAT care team, and previous studies have shown that patients acknowledged the importance of both [19].

Although shown to be effective and well-received [17,18,19], the TREAT study revealed that patients are unfamiliar with team-based care, particularly when provided in a telemedicine format. Specifically, a need was identified to help prepare patients to engage in discussions about glycemic management and introduce them to the various members of the TREAT team. Building on the TREAT model, the purpose of this feasibility project was to create, integrate and evaluate a SDM approach that could be used in this telemedicine model of care. Goals included enhancing the patient’s understanding of key outcome measures such as HbA1C, and enhancing the patient’s understanding of the roles of different providers in the TREAT model. Additionally, the project sought to increase patient engagement in medical decisions, thereby improving team communication and incorporating the patient more fully into the care team. 

## 2. Methods

### 2.1. Recruitment and Setting

Patients with type 2 diabetes mellitus (T2D) residing in a medically underserved rural community, and referred by their local PCP and scheduled for a TREAT telemedicine glycemic consultation at a rural hospital clinic as part of usual practice were invited to participate in this project. This project was approved as a Quality Improvement project (Project #0001366) by the UPMC Quality Review Committee.

### 2.2. Intervention

**Figure 1:**
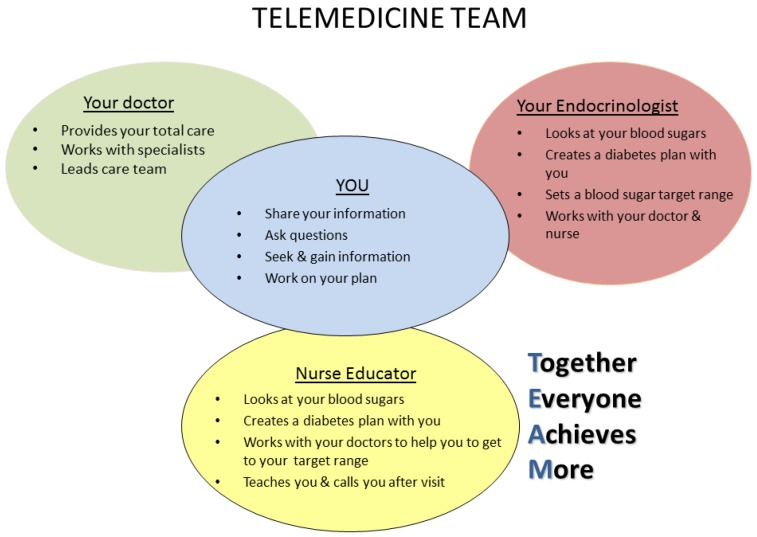
The TREAT team.

**Figure 2:**
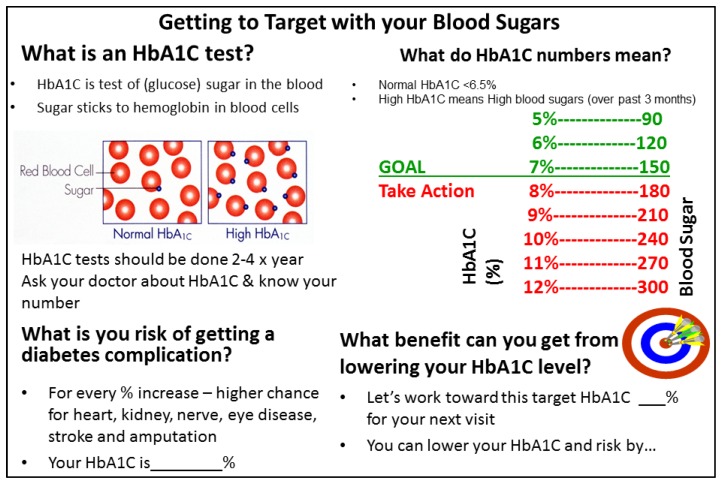
HbA1c decision aid.

SDM Aids: To help inform and engage patients in the TREAT process, two SDM aids were developed. The first aid was designed to familiarize patients with the TREAT delivery model and a team-based approach to care. It included a brief description outlining the telemedicine visit and graphically highlighting the roles of each TREAT team member, which included the referring PCP, endocrinologist and DE as well as the patient (Figure 1). The second aid, shown in Figure 2, centered on glycemic control, specifically addressing HbA1c since it is central to glycemic management (the focus of the TREAT visits). Key elements of SDM were integrated into the HbA1c aid including providing information in a simple and meaningful way, describing risks and benefits, and incorporating tools to enable patients to evaluate their options and make decisions. In this case, decisions related to setting an HbA1c target goal and determining behavioral strategies to help meet that goal.

SDM Training: Providers were trained on how to incorporate the content and SDM approach into current practice with the goal of empowering the patient to be an active participant. Scripts were developed to guide discussions about SDM aids with patients.

**Figure 3:**
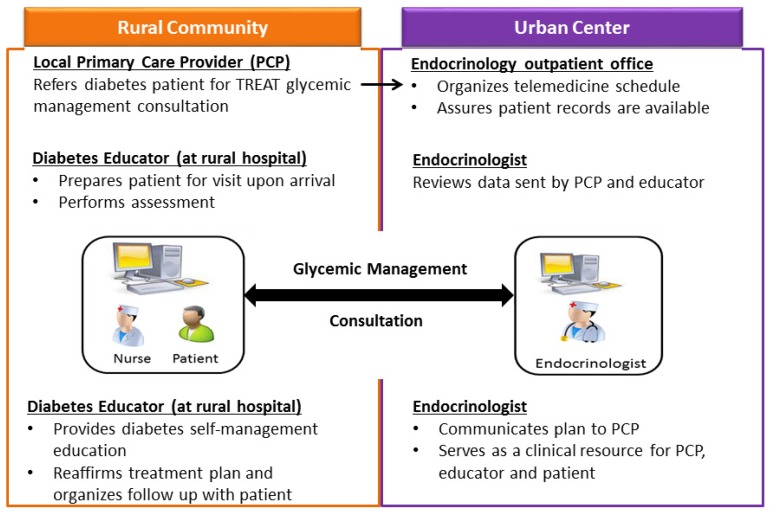
The telemedicine for reach, education, access and treatment (TREAT) glycemic management process.

SDM Process: The SDM process, depicted in Figure 3, was initiated by mailing SDM aids to the patient when the telemedicine visit was scheduled. Prior to the TREAT consult, the diabetes educator called the patient to encourage him/her to familiarize themselves with the SDM aids, consider the information contained within, and answer any questions about the upcoming visit. During the TREAT consult, the HbA1c decision aid was used as a supplement and guide to help the patient feel comfortable with the structure of the visit and empowered to be an active participant. The aid was also used by the endocrinologist to collaboratively set an HbA1c target goal with the patient. At the end of the visit, the DE reviewed the experience with the patient and used the HbA1c tool to help guide the patient in identifying a behavioral goal to support their HbA1c target. The DE then prepared a take home summary that included HbA1c and behavioral goals and the patient’s decisions regarding the next steps in his/her treatment plan. The patient’s behavioral goal was included in the office visit note and transmitted to the PCP. On return visits, care was taken to discuss progress with the behavioral goal early in the visit.

### 2.3. Evaluation

A feasibility project occurring over a twelve month period was conducted to evaluate patient and provider experiences with the SDM approach. During the project period, 17 patients (12 females, 5 males) with T2D participated in TREAT visits; 12 returned for at least one quarterly follow-up visit. Patient behavioral goals established at the end of the TREAT visits were reviewed and patient and provider feedback about their experiences was obtained. In addition, HbA1c (extracted from electronic medical records) and diabetes self-care behaviors (measured with the Summary of Diabetes Self-Care Activities measure (SDSCA)) [20] were assessed prior to the initial TREAT visit and, on average, six months after baseline for patients with follow-up appointments during the study period. The self-administered SDSCA questionnaire was used to further examine the benefits associated with the diabetes education portion of the intervention.

### 2.4. Data Analysis

Changes in HbA1c and SDSCA scores were examined by Wilcoxon rank sum test, which is used for small samples and non-normal distributions. A *p* < 0.05 was considered significant. All statistical analyses were performed with IBM SPSS Statistics for Windows, version 24 (IBM Corporation, Armonk, NY, USA).

## 3. Results

### 3.1. Goal Setting

All participants (*n* = 17) established diabetes self-management goals after participating in the TREAT consult, during which the HbA1c SDM aid was reviewed. Most participants chose diet-related goals. A majority of the goals directly referenced improving HbA1c and/or blood glucose levels. Examples of goals are presented in Table 1.

### 3.2. Diabetes Outcomes

Diabetes outcomes are shown in Table 2. HbA1c values significantly improved for participants with follow up visits (*n* = 12; *p* = 0.017). Diabetes self-efficacy, blood glucose monitoring, and adherence to diabetes medication (insulin or oral medications) were reportedly high at baseline and remained so for approximately 6 months after the initial TREAT visit. Other self-care measures also remained unchanged with the exception of consumption of high fat foods, which significantly decreased during the project period.

### 3.3. Provider and Patient Feedback

Endocrinologists stated that they were initially skeptical of the SDM approach and concerned that patients would not be receptive to it. However, endocrinologists and DEs reported positive experiences and were impressed by the level of patient engagement. Specifically, providers stated that the SDM aids increased cohesion among team members (including patients), were useful in communicating meaning of HbA1c levels, benefits, and risks of glucose control and helped facilitate conversation and behavioral goal setting. Patients expressed a better understanding of diabetes and a feeling of being empowered.

## 4. Discussion

This project built upon the previously established TREAT care model to develop and integrate a SDM tool. Patients were introduced to the care team and the expectations of team members, including patients, via descriptions and graphics prior to the first telemedicine visit. They were also provided with background education on the importance of HbA1c prior to the first meeting, then were engaged in the process of establishing goals within the shared decision-making framework.

Simple educational aids were shown to be useful in preparing patients for a potentially new experience with SDM, and that the process was well-accepted by patients. Members of the TREAT team reported that they were unaware that patients had little understanding in regards to interpreting HbA1C values. They found the SDM aids and process to be useful in supporting knowledge and communication.

Additionally, this project demonstrated that SDM could be integrated into the workflow of a team visit with good provider and patient satisfaction. Patient satisfaction is increasingly seen as a critical measure of success in health care. By communicating the patients’ tangible self-care goals to their PCP within the assessment and plan, we provided an opportunity for reinforcement during other health care visits, without increasing the burden of visit documentation. Given the chronic nature of diabetes and ongoing need for support by all members of the care team, increased communication of specific care goals may promote and sustain patient adherence and self-care.

Similar to this program, another diabetes SDM program specifically tailored for an underserved African American population included a team-based and educational approach. Participants reported high satisfaction and improved knowledge, along with improvement in decision-making self-efficacy scores, however these changes were not statistically significant [21]. While participants in the TREAT SDM intervention experienced improved knowledge and glycemia [19], except for a reduction in consumption of high fat foods, self-care outcomes were also unchanged in this rural study population. 

Limitations of our feasibility project included a relatively small sample size. Sample size was limited by the availability of telemedicine appointments within the study period (typically 3–4 new patient appointments per month). The project was not designed to evaluate the effect of the SDM process on outcomes such as HbA1c with as much rigor as the original TREAT study [18]. Additionally, longer follow-up is needed to fully evaluate effects on HbA1c and other diabetes outcomes with continued care.

While SDM approaches have shown to be beneficial in engaging patients in making informed health care decisions, there is limited evidence that SDM influences clinical and behavioral outcomes [22]. The concept of patient involvement (SDM; informed collaborative choice) is emerging in the literature and requires an accurate and comprehensive assessment of all of the clinical, behavioral and psychosocial themes respective of the specific disease state.

While SDM enhanced our team visit process via telemedicine, these tools are adaptable to other care environments. Additionally, the telemedicine team approach can be a platform for testing of other patient care tools. Overall, these are encouraging findings. As the health care landscape changes and patients are also treated as “consumers” and expected to make informed choices for their health care, tools that incorporate SDM are even more important.

## Figures and Tables

**Table 1:** Examples of diabetes self-management goals.

Initial HbA1c Goal	Detailed Behavioral Goal
HbA1c < 7% by making healthy food choices	Increase healthy food choices and limit carbohydrates with meals; limit sweets to once a week.
Lower HbA1c to 7.5%	Cut down on snacks at bedtime.
Lower HbA1c by 1 point to 7.5% by eating healthy and monitoring blood sugar	Follow Weight Watchers; check blood sugar more routinely.

**Table 2:** Diabetes outcomes.

	Before Initial Telemedicine Visit	~6 Months Post Visit	*p*-Value
HbA1c (% ± standard deviation)	10.1 ± 1.4	8.6 ± 1.2	<0.05
Diabetes Self-Care Measures (days/week) ^a^			
Adhere to general diet	4.6 ± 1.9	4.4 ± 1.2	ns
Consume 5+ servings fruit & vegetables	4.3 ± 2.1	4.2 ± 1.9	ns
Consume high fat foods	4.4 ± 2.4	2.7 ± 1.7	<0.05
Monitor blood glucose	5.3 ± 2.3	6 ± 1.4	ns
Adhere to insulin or oral medications	7	6.4 ± 2.1	ns

ns = not significant; ^a^ Range: 1 to 7 days per week.
